# Single-cell resolution reveals RalA GTPase expanding hematopoietic stem cells and facilitating of BCR-ABL1-driven leukemogenesis in a CRISPR/Cas9 gene editing mouse model

**DOI:** 10.7150/ijbs.76993

**Published:** 2023-02-13

**Authors:** Zhao Yin, Rui Su, Lanlan Ge, Xiuyuan Wang, Juhua Yang, Guiping Huang, Chuting Li, Yanjun Liu, Keda Zhang, Lan Deng, Jia Fei

**Affiliations:** 1 Department of Biochemistry and Molecular Biology, Medical College of Jinan University, Guangzhou 510632, China; 2 Department of Hematology, Guangdong Second Provincial General Hospital, Jinan university, Guangzhou 510317, China; 3 Guangdong Engineering Technology Research Center of Drug Development for Small Nucleic Acids, Guangzhou, China; 4 Guangzhou Antisense Biopharmaceutical Technology Co., Ltd., Guangzhou 510632, China; 5 Center Lab of Longhua Branch and Department of Infectious Disease, Shenzhen People's Hospital (The Second Clinical Medical College, Jinan University), Shenzhen, Guangdong 518020, China; 6 Department of pathology (Longhua Branch), Shenzhen People's Hospital (The Second Clinical Medical College, Jinan University), Shenzhen, Guangdong 518020, China; 7 College of Pharmacy, Shenzhen Technology University, Shenzhen 518118, China; 8 Department of Hematology, Ninth People's Hospital, Shanghai Jiao Tong University School of Medicine, Shanghai 200011, China

**Keywords:** RalA/single-cell resolution, leukemia stem cells, normal hematopoietic stem cells, imatinib resistance, mouse model with gene editing

## Abstract

BCR-ABL oncogene-mediated Philadelphia chromosome-positive (Ph+) chronic myeloid leukemia (CML) is suggested to originate from leukemic stem cells (LSCs); however, factors regulating self-renewal of LSC and normal hematopoietic stem cells (HSCs) are largely unclear. Here, we show that RalA, a small GTPase in the Ras downstream signaling pathway, has a critical effect on regulating the self-renewal of LSCs and HSCs. A RalA knock-in mouse model (RalA^Rosa26-Tg/+^) was initially constructed on the basis of the Clustered Regularly Interspaced Short Palindromic Repeats/Cas9 (CRISPR/Cas9) assay to analyze normal hematopoietic differentiation frequency using single-cell resolution and flow cytometry. RalA overexpression promoted cell cycle progression and increased the frequency of granulocyte-monocyte progenitors (GMPs), HSCs and multipotent progenitors (MPPs). The uniform manifold approximation and projection (UMAP) plot revealed heterogeneities in HSCs and progenitor cells (HSPCs) and identified the subclusters of HSCs and GMPs with a distinct molecular signature. RalA also promoted BCR-ABL-induced leukemogenesis and self-renewal of primary LSCs and shortened the survival of leukemic mice. RalA knockdown prolonged survival and promoted sensitivity to imatinib in a patient-derived tumor xenograft model. Immunoprecipitation plus single-cell RNA sequencing of the GMP population confirmed that RalA induced this effect by interacting with RAC1. RAC1 inhibition by azathioprine effectively reduced the self-renewal, colony formation ability of LSCs and prolonged the survival in BCR-ABL1-driven RalA overexpression CML mice. Collectively, RalA was detected to be a vital factor that regulates the abilities of HSCs and LSCs, thus facilitating BCR-ABL-triggered leukemia in mice. RalA inhibition serves as the therapeutic approach to eradicate LSCs in CML.

## Introduction

Leukemic stem cells (LSCs) and hematopoietic stem and progenitor cells (HSPCs) have self-renewal ability. Among them, LSCs indefinitely propagate leukemia, while normal HSCs can sustain multidirectional differentiation 1, 2. Because LSCs and hematopoietic stem cells (HSCs) share certain similar functions, they may be modulated through similar pathways 3, 4. Thus, more knowledge of the mechanisms regulating HSC function can help to understand the functions of both HSCs and LSCs 5.

Chronic myelogenous leukemia (CML) is derived from the neoplastic transformation of HSCs 6, 7. BCR-ABL1 transforms HSCs into CML stem cells (referred to here as LSCs), which then generates the development of a clonal myeloproliferative disease 8. Therefore, the important regulators of the self-renewal of HSCs must be considered.

As small GTPases, Ras proteins play various roles of molecular switches, which can transduce signals from their activated receptors to the downstream effectors for regulating cell growth, self-renewal, cell differentiation, and cell survival. Over one-third of cancers in humans carry active Ras mutations 9. The BCR-ABL1 oncoprotein can constitutively activate the Ras-associated pathways in CML. Moreover, in the BCR region, tyrosine 177 autophosphorylation can recruit growth factor receptor-bound protein 2 (GRB2, an adaptor protein) through the SH2 domain 10, which further binds SOS (Ras' guanine nucleotide exchange factor [GEF]) leading to Ras activation 11.

Apart from the Wnt/β-catenin pathways 12, the Ras-related signaling pathways also have a critical effect on cancer stem cells (CSCs) in CML 13. Ras combines with various adaptor/effector proteins to activate diverse pathways, including the PI3K/mTOR signaling pathway. The PI3K/mTOR pathway inhibitors sensitize CML LSCs 14.

Extensive efforts have been made to develop Ras inhibitors for antitumor treatment. However, treatments aiming to interfere with Ras modification post-translationally 15 resulted in dismal outcomes. Consequently, researchers are seeking to target the downstream components of Ras, such as RalA.

Ras can combine with and activate Ral-specific GEFs (RalGEFs), which further activate RalA. RalA has critical effects on *in vitro* anchorage-independent growth and *in vivo* tumorigenic growth in mice. Over the past decade, several studies have reported that Ral proteins, together with their corresponding effectors, have important effects on oncogenesis 16. RalA activation plays an important role in Ras-mediated human cell carcinogenesis 17; specifically, the RAL family proteins have critical effects on BCR-ABL1-induced leukemogenesis 18. Our previous studies revealed that RalA could play a role of an oncogene in CML 19. Thus, RalA is a critical regulator of hematopoiesis; nevertheless, the precise role of RalA remains poorly understood. Because of the rapidly developing single-cell RNA sequencing (scRNA-seq), it is possible to determine cellular taxonomy and tag differentiation and reveal the single-cell transcriptional networks 20, 21.

Here, we constructed Rosa26-targeted RalA knock-in (KI) mice and used single-cell resolution with flow cytometry (FCM) to assess the effect of RalA on hematopoietic regulation. By using this strategy, we revealed that RalA GTPase influenced the self-renewal of normal and malignant HSPCs and facilitated BCR-ABL-driven leukemogenesis by interacting with RAC1 in a mouse model constructed with CRISPR/Cas9 gene editing technology. Our results help to understand the effect of RalA on regulating LSC maintenance of HSPCs and CML.

## Materials and Methods

### Mouse models

C57BL/6 mice were obtained from Biocytogen Laboratory Animal Co. (Beijing) and maintained in the Jinan University Animal Facility, Guangzhou, China. Each mouse was housed in a sterile environment, with unlimited access to food and water.

Each animal experiment was conducted strictly in accordance with relevant animal care guidelines. The study protocols were approved by the Jinan University Institutional Animal Care and Use Committee and Biocytogen (No. 110360204018814185).

### Rosa26-targeted RalA KI mice developed from C57BL/6 mice based on CRISPR/Cas9

A web-based software (http://crispor.tefor.net/) was used for designing single-guide RNAs (sgRNAs). A specific allele site was chosen as the target sequence in the mouse Rosa26 locus at its introns 1 and 2, which was sgRNA: AAGGCCGCACCCTTCTCCGGAGG. The mRNAs of sgRNA and Cas9 were generated through *in vitro* transcription (IVT). GenScript Biotechnology prepared the target donor DNA with the homology arms to homologously recombine and express elements of hRalA (GenBank No. NM_005402.4). The target fragment was homologously recombined with the pUC57Kan-R26 vector. C57BL/6 female mice (4 weeks old) were intraperitoneally (i.p.) administered serum gonadotropin from a pregnant mare and human chorionic gonadotropin (HCG, 10 U/mouse) after 48 h, followed by immediate mating with C57BL/6 male mice (12 weeks old). Fertilized embryos were harvested the next morning, followed by microinjection of 50 ng/mL Cas9 mRNA, 50 ng/mL sgRNA, and 10 ng/mL hRalA-targeting construct into the fertilized embryo cytoplasm by adopting the standard microinjection system (Transfer Man 4r; Eppendorf, Germany). Subsequently, we collected the surviving eggs and cultivated them overnight at 37 °C with 5% CO_2_. On the following day, eggs were placed into the oviducts of pseudo-pregnant ICR mice to obtain live pups. Next, each obtained mouse was examined for the hRalA gene by PCR analysis, with genomic DNA (gDNA) from tail-biopsied samples as a template. These mice were crossed with CMV-Cre mice to acquire RalA-overexpressing mice (Supplementary Figure S1).

### FCM analysis

In terms of stem cell analysis, mouse bone marrow cells (BMCs) were collected from C57BL/6 mice (6 weeks old) and subjected to staining. BMCs were extracted and subjected to lysis of red blood cells (RBCs), followed by incubation with antibodies shown below. Later, splenic tissue and BMC-derived single-cell suspensions were prepared, followed by staining on ice for 20 min. The following HSPC markers were used: LT-HSC: Lin- (PerCP-Cy™5.5); FVS780- (APC-Cy™7); C-KIT+ (CD117, APC); Sca+ (PE-Cy™7); CD34- (FITC); Flk2- (BV421); GMP: Lin- FVS780- C-KIT+ Sca- CD34+ CD16/32+ (FcγRII, BV421); MPP: Lin- FVS780- C-KIT+ Sca+ CD34+ Flk2+; Megakaryocyte-erythroid progenitor (MEP): Lin- FVS780- C-KIT+ Sca- CD34- CD16/32-; Common myeloid progenitor (CMP): Lin- FVS780- C-KIT+ Sca- CD34+ CD16/32-; GR-1 (Ly-6G and Ly-6C) and MAC-1(CD11b); B cell (CD19); and T cell (CD90.2). All antibodies used in FCM were provided by BD Biosciences (New Jersey, USA). Cells were stained, examined, and sorted using FACS verse and FACS AriaII (Becton Dickinson). Flowjo software (TreeStar, Inc.) was used for FCM data analysis.

### scRNA-Seq

BMCs were harvested from 16-week-old C57BL/6 mice. CD117+ cells were obtained with CD117 microbeads (Miltenyi Biotec) using the Lineage Cell Depletion Kit (mouse). The 10x Genomics Single Cell 3′ v2 Reagent Kit was used to prepare samples according to specific instructions. The cells were then counted, and approximately 6000 cells from every sample were loaded onto a 10x Genomics single-cell-A chip to construct the cDNA library in accordance with the instructions of the Single Cell 3′ Reagent Kit v3 after some modifications of PCR cycles based on the determined cDNA level (recommendations from 10X Genomics). Each sample was then combined, followed by normalization to 10 nM and dilution with an elution buffer containing 0.1% Tween 20 (Sigma) to 2 nM. Later, Novaseq 6000 was used for sample sequencing with the following parameters: Read 1-26 cycles, read 2-98 cycles, and index 1-8 cycles. Each sample was sequenced to the median depth of 50,000 reads/cell.

### Real-time quantitative Polymerase Chain Reaction (RT-qPCR)

We collected RalA^Rosa26-Tg/+^ and RalA^+/+^ bone marrow that CD117+ cells were obtained by Lineage Cell Depletion Kit (mouse) and CD117 microbeads, and the TRIzol method (Takara) was adopted for extracting total RNA, which was later prepared into cDNA 5 × qRT SuperMix (All-in-one cDNA Synthesis SuperMix, Takara). SYBR-Green RT-PCR was then conducted to detect specific mRNA. The ΔΔCt approach was utilized to determine knockdown efficiency, with β-actin as the endogenous reference.

### Proliferation Assay

Each mouse was i.p. administered 150 mg/kg BrdU (Sigma). After 6 h, splenocytes and BMCs were harvested for resuspension in Iscove's modified Dulbecco's medium (IMDM; Invitrogen) containing 20% FBS. After the lysis of RBCs, PBS was added to rinse the remaining cells. Next, 1% paraformaldehyde (PFA) was added to fix the cells overnight, followed by treatment with 0.01% Tween 20 at 4 °C; the cells were then washed twice and resuspended in DNase (Sigma) for 30 min at 37 °C. The cells were later rinsed twice, followed by staining for 20 min with anti-BrdU-FITC (Pharmingen). After washing twice, FCM was conducted for cell analysis.

### Generation of retroviral stocks

Plat-E cells were transiently transfected with a retroviral construct murine stem cell virus (MSCV)-BCR-ABL1-internal ribosome entry site (IRES)-enhanced green fluorescent protein (GFP) in order to generate high-titer helper-free retroviruses, as described previously 22.

### CML-like leukemia model and transplantation assay

BMCs were cultured in IMDM with GlutaMAX including 20% FBS, recombinant mouse stem cell factor (SCF, 50 ng/mL) (PeproTech, USA), 2-mercaptoethanol (50 µM), 1% penicillin/streptomycin (PS), recombinant mouse interleukin (IL)-3 (10 ng/mL), and IL-6 (25 ng/mL) or subjected to retroviral transformation with BCR-ABL1. Six- to 8-week-old wild-type (WT) C57BL/6 mice and RalA^ Rosa26-Tg/+^ mice were used for leukemia genesis assays. CML was induced as described previously 23. Briefly, to construct the CML model, BCR-ABL1 retrovirus was transfected into BMCs from donor mice treated with 200 mg/kg 5-fluorouracil (5-FU) through co-sedimentation with IL-3/IL-6/SCF. RalA^+/+^ recipient mice were irradiated with 1100 cGy rays (administered as two separate doses of 550 cGy) and injected with CML cells (0.5 × 10^6^) through the tail vein. The recipient mice were injected with BCR-ABL1-transfected donor BMCs (5 × 10^5^) in RalA^+/+^ or RalA^ Rosa26-Tg/+^ mice. Each CML cohort contained 8 mice.

### G-LISA™ assay

After the lysis of mouse BMCs with lysis buffer containing a protease inhibitor cocktail (1×), the G-LISA™ Ral Activation Assay Biochem Kit™ (Cytoskeleton, Inc., Denver, CO) was used to measure the activity of RalA GTPase in accordance with specific protocols. In brief, the lysates were subjected to 20 min incubation in the Ral-GTP affinity plate. Following incubation, the solution in each well was discarded, and each well was rinsed twice with washing buffer at ambient temperature. Next, an antigen-presenting buffer was supplemented to each well at room temperature by using a multichannel pipettor, followed by incubation for 2 min at ambient temperature. The antigen-presenting buffer was discarded, and each well was subjected to 45 min incubation with diluted anti-RalA primary antibodies at ambient temperature with the orbital microplate shaker (400 rpm). Each well was rinsed again, followed by another 45 min incubation with diluted secondary antibodies at ambient temperature and followed by 15 min incubation with horseradish peroxidase (HRP) detection reagent at ambient temperature. In addition, the reaction was terminated by the addition of HRP stop buffer, and absorbance (OD) values were measured with a spectrophotometer (EPOCH2T, Biotek, USA) using a microplate reader at 490 nm.

### Cell viability assay

The 3-(4,5-dimethylthiazol-2-yl)-2,5-diphenyltetrazolium bromide (MTT) assay was performed to determine cell viability. Mouse BMCs (1 × 10^4^/well) transfected with the BCR-ABL retrovirus were seeded onto 96-well plates, followed by treatment with imatinib or ponatinib at the dose of 0-300 nmol/L for 48 h at 37 °C. Next, MTT (final concentrations: 0.5mg/mL) was added to each well for 4 h and 150µL DMSO were added into each well. Finally, a microplate reader (EPOCH2, Biotek) was used to measure OD values at 570 nm.

### Lentivirus transfection into CML CD34+ cells

CML BM mononuclear cells (BMMCs) were acquired from adult donors at the Guangdong Second Provincial General Hospital and Guangdong Provincial Emergency Hospital. Informed consent was obtained from all donors for sample collection, which was performed in line with the Declaration of Helsinki and guidelines from the institutions. In brief, the positive magnetic bead sorting protocol (StemCell Technologies, USA) was used for isolating CD34+ cells. 293T cells were cotransfected using a RalA-targeting shRNA or a control shRNA (Scramble) and a pCMV-VSVG envelope construct or a pCMV-dR8.2 packing construct to produce lentiviruses. Virus-containing supernatants were collected following centrifugation of CML CD34+ cells (1 × 10^6^ cells/mL) for 90 min at 1500 × *g* at 32 °C for 2 cycles. After 48 h, the cells were collected for subsequent experiments.

### Clone forming assay

In brief, BCR-ABL1-transfected mouse CML-like cells (1 × 10^5^) were used for methylcellulose clone forming assays. The mouse MethoCult medium (StemCell Technologies) was added to resuspend cells, followed by culturing of the cells on the plates (diameter, 3 cm). Evaporation was prevented by adding phosphate buffer saline (PBS) to the plate. After 14 days, the number of formed clones was determined.

### Immunoprecipitation and LC-MS assays

After pelleting of K562 cells at 1500 × *g* for 1.5 min, the cells were resuspended and lysed with the lysis buffer containing 137 mM NaCl, 20 mM Tris, 350 µL, 2 mM CaCl_2_, 1% Triton X-100, and 2 mM PMSF. The lysates were collected, clarified for 20 min at 16,000 × *g*, and probed overnight using RalA antibodies, followed by the addition of protein A/G beads (Santa Cruz Biotechnology, Dallas, TX). After the reaction was completed, lysis buffer was added to rinse beads thrice, and SDS-PAGE was carried out to elute bound proteins. The eluted proteins were visualized by western blotting analysis and LC-MS. To analyze the RalA pathway, the Kyoto Encyclopedia of Genes and Genomes (KEGG, http://www.genome.jp/) analysis was performed to confirm the proteins identified from RalA immunoprecipitates.

### Data analysis

Data are expressed as mean ±SD. For independent samples, P<0.05 in a two-tailed t-test was regarded to be of statistical significance. Log-rank test was used to analyze Kaplan-Meier (KM) survival curves. GraphPad Prism software (San Diego, CA) was employed to perform statistical analysis.

## Results

### Generation of RalA KI mice and RalA GTPase activity analysis

**Figure 1:**
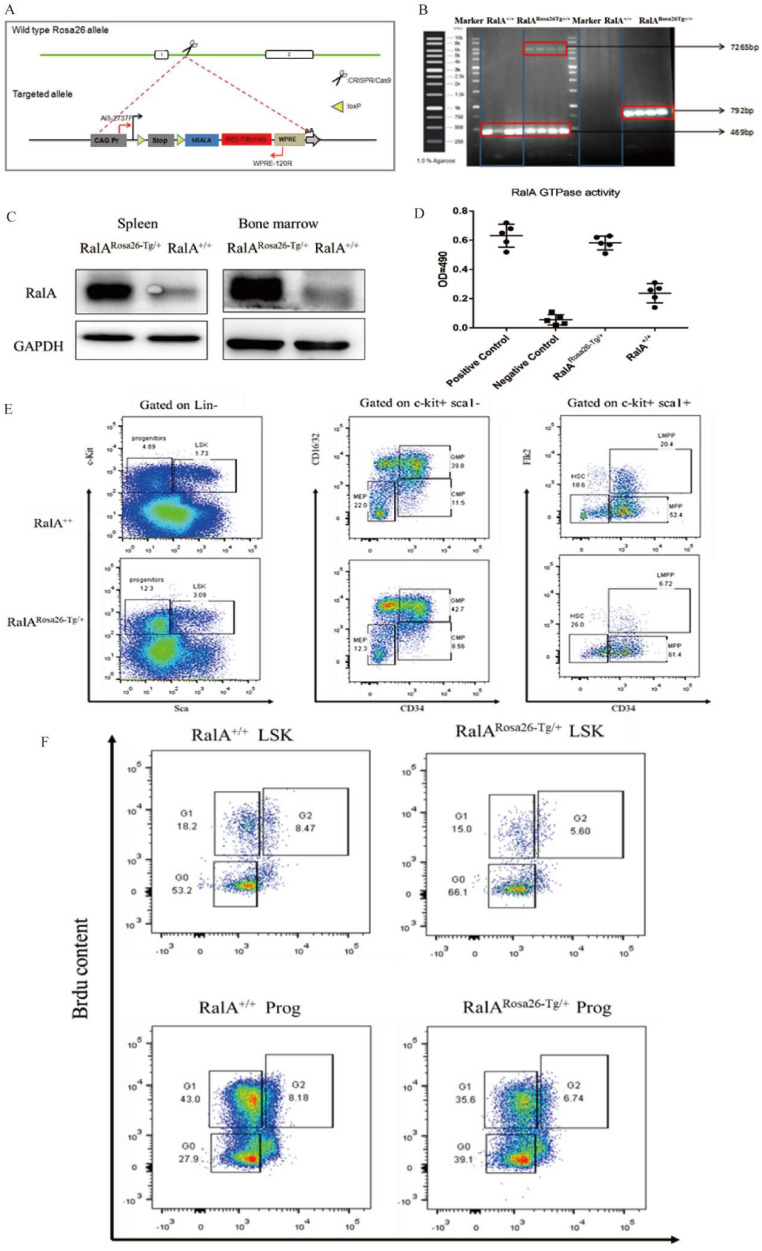
** Overexpression of RalA increases self-renewal of hematopoietic stem cell compartment and granulocyte-macrophage progenitors in RalA^Rosa26-Tg/+^ mice.** (A) Diagram of CRISPR/Cas9-mediated KI of the *hRalA* gene into the Rosa26 locus. An expression cassette with the CAG promoter and the *hRalA* gene was inserted between the homologous arms at the Rosa26 locus through the CRISPR/Cas9 system. Gene editing was achieved by homology-directed repair (HDR) using the repair template vector for precise insertion of the new sequence. (B) Expression of full-length hRalA in mice transfected with donor, hRalA, and Tag Tdtomato, a 7265-bp DNA fragment containing the entire coding sequence of hRalA. Tdtomato was amplified with the primers Rosa-F and Rosa-R, while normal controls could not amplify the 7265-bp DNA fragment. The 792-bp fragment was also a specific DNA fragment in RalA^Rosa26-Tg/+^ mice. (C) Immunoblot analyses of RalA were performed in RalA^Rosa26-Tg/+^ and RalA^+/+^ mice spleen and bone marrow (BM). GAPDH was used as a loading control. Western blotting analysis showed that RalA was overexpressed at the protein level in RalA^Rosa26-Tg/+^ mice. (D) Total RalA activation levels in RalA^Rosa26-Tg/+^ and RalA^+/+^ mice BM cells determined by G-LISA assay. G-LISA assay showed GTPase activity was also significantly higher in RalA^Rosa26-Tg/+^ mouse BM samples as compared to that in RalA^+/+^ mouse BM samples. (E) RalA regulates murine HSC self-renewal and differentiation. Flow cytometric analysis of LSK (Lin-Sca1+c-kit+), LT-HSC (LinSca1+c-kit+CD34-Flk2-), and MPP (LinSca1+c-kit+CD34+Flk2+) cells in the BM from controls; flow cytometric analysis of myeloid progenitors, including CMP (Lin-Sca1-c-kit+CD34+CD16/32+low), granulocyte-macrophage progenitors (GMP) (Lin-Sca1-c-kit+CD34+ CD16/32+high), and MEP (LinSca1-c-kit+ Sca-CD34-CD16/32-) in the BM from RalA^Rosa26-Tg/+^ mice. The GMP population was significantly increased in RalA^Rosa26-Tg/+^ mice as compared to that in RalA^+/+^ mice. (F) The cell cycle analysis of LSK cells using DAPI and BrdU showed an accumulation of G0+G1 phase cells in RalA^Rosa26-Tg/+^ mice.

For generating hRalA-expressing KI mice, the construct that contained hRalA cDNA was cloned in the mouse Rosa26 locus with the CRISPR/Cas9 system by homologous recombination (Figure 1A). Briefly, IVT was conducted to prepare guide RNA, while CRISPOR software was used for analyzing the possible off-target mutations. Four- to 6-week-old B6 female mice were superovulated and fertilized with sperm cells collected from 10- to 12-week-old B6 male mice to prepare fertilized eggs. The B6 mouse Rosa26 locus-targeting CRISPR/Cas9 machinery was then microinjected into female mice. The target construct was co-injected together with the specific Cas9 mRNA and guide RNAs. The ICR outbred, cultured mice were later used for the development of engineered eggs, followed by genotyping with neonate mouse tail tip DNA. Microinjection of fertilized eggs was then conducted to obtain F1 mice, which were later crossed with CMV-Cre male mice for obtaining the RalA^Rosa26-Tg/+^ progeny (Supplementary Figures S2 and S3). For validating the gene-specific targeting-based genome editing, PCR was conducted to analyze the gDNA collected from tail biopsy samples, as shown in Figure 1B. The 7265-bp band was the specific band inserted in the Rosa26 locus. Because RalA^Rosa26-Tg/+^ mice contained a tomato tag, red skin was formed that enabled easy identification (Supplementary Figure S4). RalA level and activity in RalA^Rosa26-Tg/+^ and RalA^+/+^ were tested in mouse BM. The RalA protein was overexpressed in the spleen and BM of RalA^Rosa26-Tg/+^ mice than in those of RalA^+/+^ mice (Figure 1C). GTPase activity was also significantly higher in RalA^Rosa26-Tg/+^ mouse BM samples than in RalA^+/+^ mouse BM samples (Figure 1D). On the basis of the above findings, successful hRalA gene targeting at the Rosa26 locus was achieved with the CRISPR/Cas9 system. RalA^Rosa26-Tg/+^ mice had a higher expression of the RalA protein and GTPase activity than RalA^+/+^ mice.

### An integrated analysis of self-renewal and differentiation of HSPCs in RalA^Rosa26-Tg/+^ mice

FCM was conducted to assess how RalA overexpression affected HSPC compartments in RalA^Rosa26-Tg/+^ mice. RalA overexpression markedly elevated the HSC-abundant LSK compartment (Lin-Sca1+c-kit+) and the corresponding subsets such as MPPs (Lin-Sca1+c-kit+CD34+Flk2+) (61.4% vs. 52.4%) and long-term HSCs (LT-HSC, Lin-Sca1+c-kit+CD34-Flk2-) (26% vs. 18.6%) in the BM. FCM results also suggested that GMPs (Lin-Sca1+c-kit+CD34+ CD16/32+) (42.7% vs. 39.8%) remarkably increased in BM (Figure 1E). Taken together, the populations of LSK, GMPs, and HSCs were increased in the BM from RalA^Rosa26-Tg/+^ mice.

Next, the current work explored the mechanism of changes in HSC activity in RalA-overexpressing mice. According to the results of BM analysis, RalA overexpression remarkably elevated the proportions of cycling progenitors and LSK cells (Figure 1E).

In RalA^+/+^ mice, we found that BrdU exposure yielded 71.4% of G0+G1 LSK cells. In contrast, the frequency of G0+G1 LSK cells was 81.1% in RalA-overexpressing mice (Figure 1F). In general, these results demonstrated that RalA maintained the nondifferentiated and quiescent state of mouse HSCs.

### Heterogeneity of single-cell transcriptomes of HSCs and GMPs

**Figure 2:**
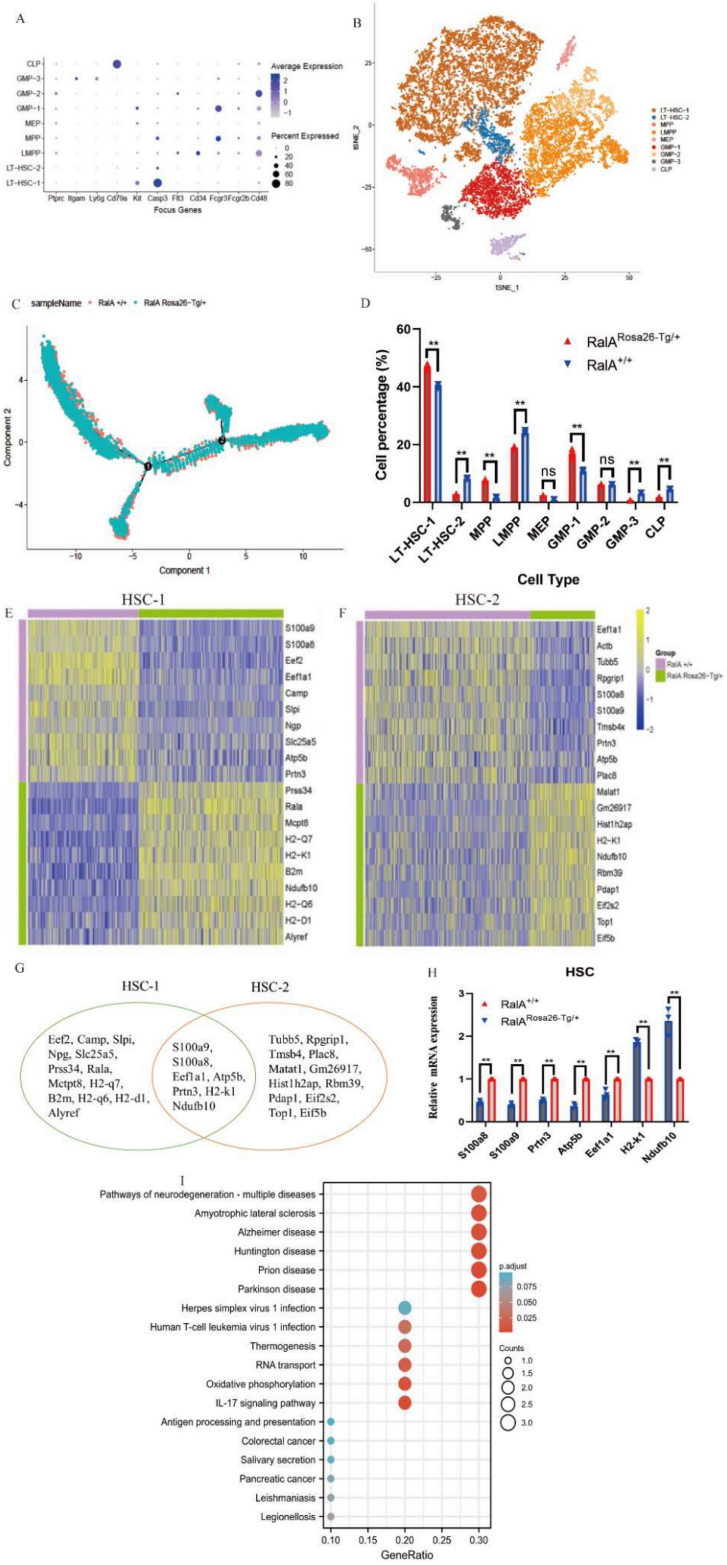
** Single-cell transcriptomes of heterogeneity in hematopoietic stem cells.** (A) Point diagram of the mean expression value of the selected marker genes for each cell type. (B) Uniform manifold approximation and projection (UMAP) clustering map of mouse BM stem cells/progenitors (c-kit+) from two integrated samples. The UMAP plot shows the distribution of the 6 cellular clusters, in which there are 2 HSC and 3 GMP subclusters color-coded according to cell subclusters. (C) The branch trajectory plot derived from the BM of RalA^Rosa26-Tg/+^ mouse and RalA^+/+^ mouse. The results revealed that the RalA^Rosa26-Tg/+^ cell type exhibited some different characteristics with RalA^+/+^. (D) Bar chart of the proportion of cell subpopulation between RalA^Rosa26-Tg/+^ and RalA^+/+^ mice. The results showed that RalA increases self-renewal of HSC1 and GMP1 cells in RalA^Rosa26-Tg/+^ mice, (n = 3), **P<0.01. Mean ±SD values of three experiments and P-values are indicated. Heatmap of the expression of top10 differentially expressed genes (E, HSC-1; F, HSC-2) (Bonferroni corrected P-value < 0.05, log_FC_> 0.25) for each group. The color scale represents the gene expression levels as a z-score. (G) In gene enrichment expression, each subcluster is displayed in the Venn diagram. (H) The differential genes are confirmed by RT-qPCR. n = 3, *P < 0.05, ** P < 0.01. Mean ±SD values of three experiments and P-values are indicated. (I) The topmost enriched terms for the upregulated DEGs in the RalA^Rosa26-Tg/+^ HSC-1 group compared to that in the RalA^+/+^ HSC-1 group, ranked according to the adjusted P-value. Benjamini-Hochberg correction for multiple hypotheses testing.

To comprehensively analyze mouse BM hematopoiesis, the present study sorted lineage-negative cells to form single-cell resolution; the marker genes are shown in Figure 2A. The UMAP clustering map of mouse BM stem cells/progenitors (c-kit+) yielded two integrated samples. The UMAP plot showed the distribution of six cellular clusters, in which there were 2 HSC and 3 GMP subclusters color-coded according to cell subclusters (Figure 2B). We then sort cells from HSCs to lineage-specified progenitors in pseudotime to understand different trajectories and development states. As shown in Figure 2C, Monocle illustrated the percentage of cell type at similar branch points. Single-cell resolution also validated the immunophenotype results wherein RalA increased the self-renewal of HSC-1, MPPs, and GMP-1 in RalA^Rosa26-Tg/+^mice (Figure 2D). Heatmaps show the expression levels of the 5 most significantly enriched genes in every cluster (Supplementary Figure S5). Next, immunophenotype-defined cellular transcriptomes were comprehensively compared. The unsupervised hierarchical clustering showed transcriptional heterogeneities of immunophenotypical HSCs (iHSCs). The iHSCs were classified into two groups with different transcriptional characteristics: HSC-1 (Figure 2E) and HSC-2 (Figure 2F).

**Figure 3:**
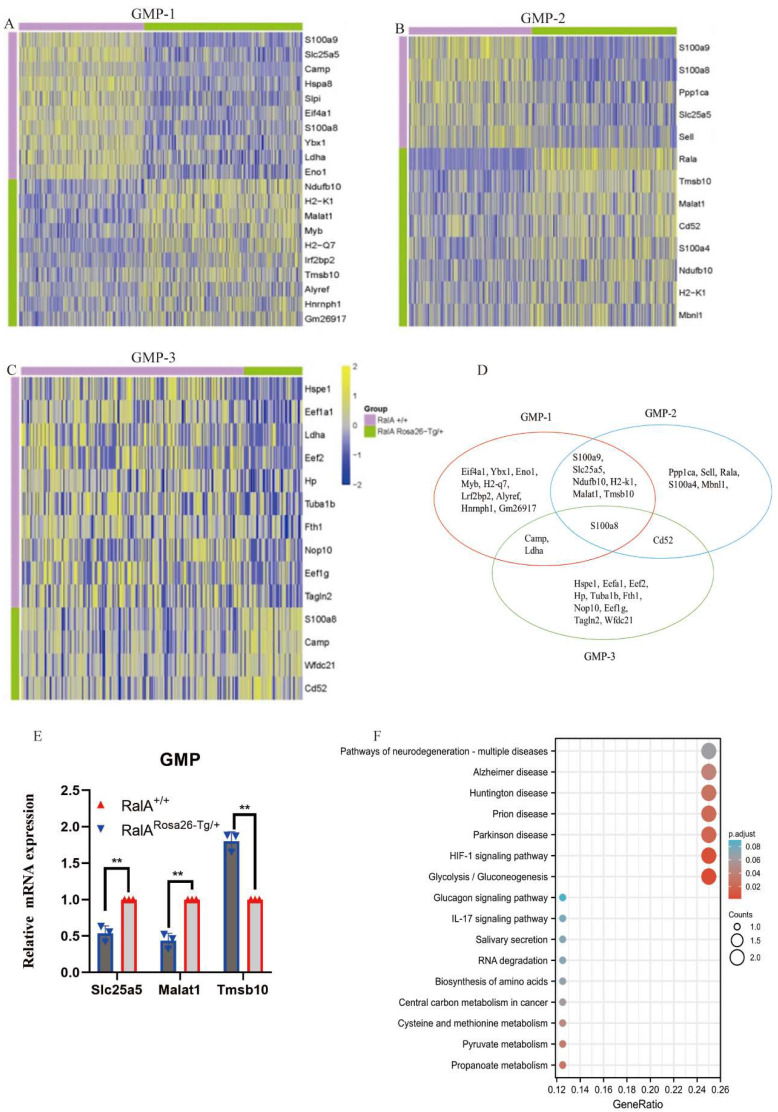
** Single-cell transcriptomes of heterogeneity in GMP cells.** Heatmap showing the expression of genes differentially expressed between RalA^Rosa26-Tg/+^ mice and RalA^+/+^ mice. Heatmap of the expression of top10 differentially expressed genes (Bonferroni corrected P-value < 0.05, log_FC_ > 0.25) for each group. The color scale represents the gene expression levels as a z-score. (A) GMP-1, (B) GMP-2, and (C) GMP-3 subclusters are displayed in the heat map. (D) Gene enrichment expression of each subcluster is displayed in the Venn diagram. (E) The differential genes between RalA^Rosa26-Tg/+^ mice and RalA^+/+^ mice in GMP cells are confirmed by RT-qPCR, n = 3, *P < 0.05, ** P < 0.01. Mean ±SD values of three experiments and P-values are indicated. (F) KEGG Biological Process terms associated with the genes significantly deregulated in RalA^Rosa26-Tg/+^ GMP-1 cells as compared to that in RalA^+/+^ GMP-1 cells, ranked according to the adjusted P-value. Benjamini-Hochberg correction for multiple hypotheses testing.

Interestingly, the top10 up- or downregulated genes were the same. *Prss34, RalA, Mcpt8, H2-k1, B2m, Ndufb10, H2-d1 H2-q6, H2-q7, and Alyref* were identified as the genes with specific and high expression in RalA^Rosa26-Tg/+^ HSC-1, while *Malat1, Gm26917, Hist1 h2ap, H2-k1, Ndufb10, Rbm39, Pdap1, Eif2s2, Top1*, and* Eif5b* were identified in RalA^Rosa26-Tg/+^ HSC-2. Seven overlapping genes were present in the top10 up- or downregulated genes between HSC-1 and HSC-2 (Figure 2G). The expression levels of *S100a8, S100a9, Prtn3, Atp5b, Eef1a1, H2-k1*,* and Ndufb10* were measured in HSPCs by real-time quantitative PCR (RT-qPCR) (Figure 2H). Figure 2I shows the results of their pathway enrichment analysis. Pathway of neurodegeneration, Amyotrophic lateral sclerosis, Alzheimer's disease, Huntington's disease, Prion disease, Parkinson's disease, Herpes simplex virus 1 infection, Oxidative phosphorylation, Human T-cell leukemia virus 1 infection, and IL-17 signaling pathway showed a close relationship with RalA expression in HSCs. By using the same strategy, the immunophenotypical GMPs (iGMPs) were classified into three groups with different transcriptional characteristics: GMP1 (Figure 3A), GMP2 (Figure 3B), and GMP3 (Figure 3C). The top10 up- or downregulated genes are shown in the heat map. *S100a8* was a unique overlapping gene among the three subclusters (Figure 3D). The expression of *Slc25a5, Malat1*, and *Tmsb10* in GMPs was confirmed by RT-qPCR (Figure 3E). Pathway enrichment analysis of the top10 up- or downregulated genes in GMP-1 cells (Figure 3F) revealed that Pathway of neurodegeneration, Alzheimer's disease, Huntington's disease, HIF-1 pathway, Prion disease, Parkinson's disease, and Glucagon pathway Glycolysis/Gluconeogenesis were enriched and involved in the RalA-regulated genes in GMPs. The results of pathway enrichment analysis of the top10 up- and downregulated genes from GMP2 and GMP3 cells are illustrated in Supplementary Figure S6A and S6B, respectively. Cell adhesion molecular binding, apoptotic signaling pathway, and neutrophil chemotaxis were related to GMP2. Moreover, GTPase activity and translational elongation contributed to GMP2. The top10 up- and downregulated genes enriched in MPP, LMPP, MEP, and CLP clusters are shown in the heat map (Supplementary Figure S7).

### RalA overexpression shortened the survival of BCR-ABL-mediated CML mice

**Figure 4:**
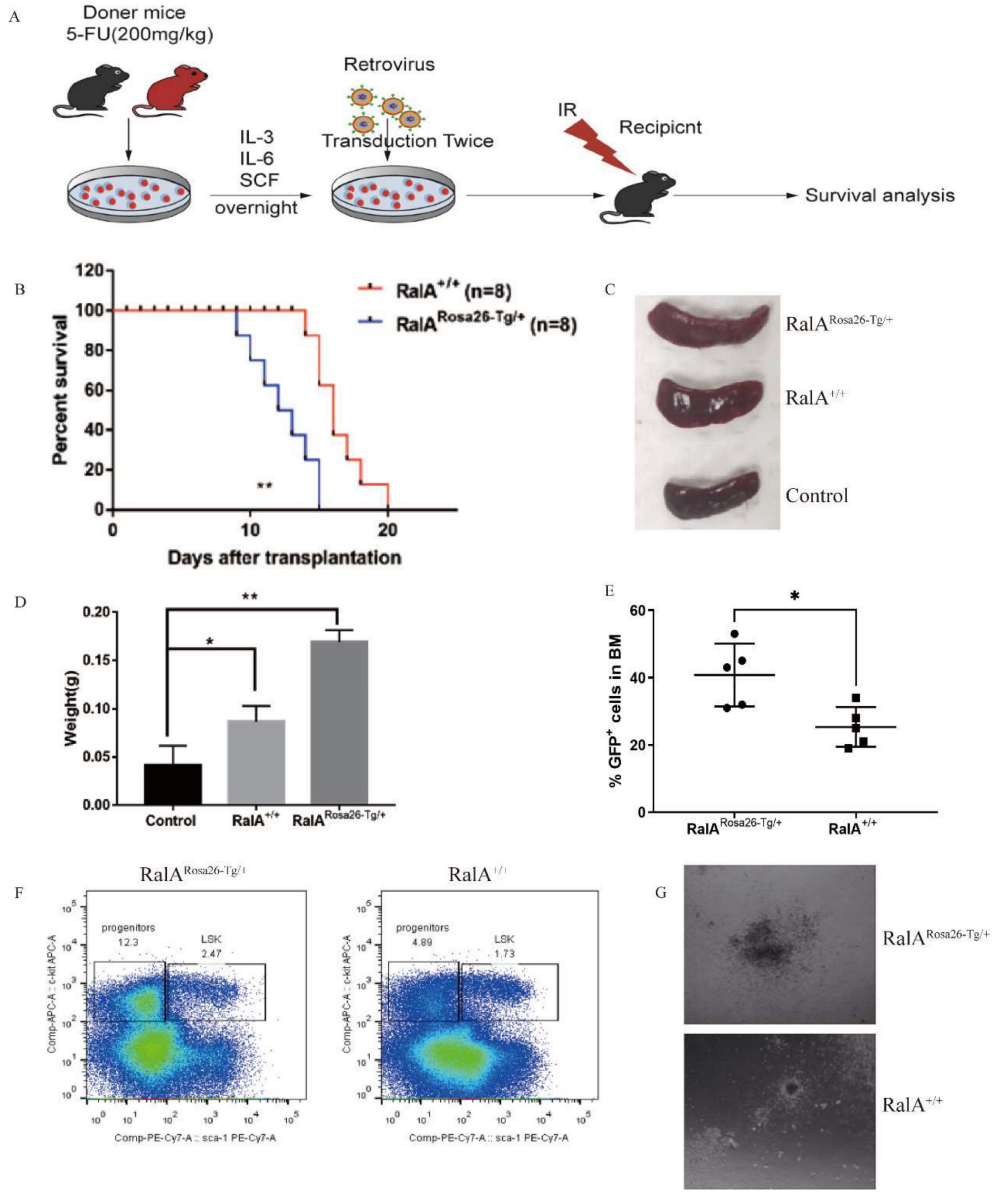
** RalA facilitates BCR-ABL-mediated CML leukemia in mice.** (A) Schematic protocol of the BCR-ABL1-driven CML model for RalA^Rosa26-Tg/+^ and RalA^+/+^ donor mice. (B) Kaplan-Meier survival curves for the recipients of BCR-ABL-transduced bone marrow (BM) cells from RalA^Rosa26-Tg/+^ and RalA^+/+^ donors. All recipients of BCR-ABL-transduced BM cells from the RalA^Rosa26-Tg/+^ donor mice developed CML and died within 2 weeks post BM transplantation, whereas the recipients of BCR-ABL-transduced BM cells from the RalA^+/+^ donor mice survived longer. (C) Gross pathology of spleens showed severe splenomegaly in the recipients of BCR-ABL-transduced RalA^Rosa26-Tg/+^ CML donor mice as compared to that in the recipients of RalA^+/+^ CML donor mice. Normal C57BL/6 mice were used as a negative control in terms of morphology. (D) The spleen showed severe splenomegaly in terms of weight in the recipients of BCR-ABL-transduced RalA^Rosa26-Tg/+^ CML donor mice as compared to that in the recipients of RalA^+/+^ CML donor mice. n = 8, *P < 0.05, ** P < 0.01. Mean ±SD values of three experiments and P-values are indicated. (E) The populations of BCR-ABL-expressing leukemia cells in BM were significantly increased in the RalA^Rosa26-Tg/+^ group. The percentages of GFP+ BM cells from the BCR-ABL-transduced RalA^Rosa26-Tg/+^ and RalA^+/+^ donor mice with CML as measured by flow cytometry. n = 5, *P < 0.05, ** P < 0.01. Mean ±SD values of three experiments and P-values are indicated. (F) FACS analysis of LSK in BM cells from RalA^+/+^and RalA^Rosa26-Tg/+^ CML mice. BMCs were isolated from RalA^+/+^and RalA^Rosa26-Tg/+^ CML mice, and the gated GFP+ cells were further analyzed by FACS to identify the Lin- population, followed by the identification of c-kit+ Sca-1+ and LSK (GFP+ Lin- c-kit+ Sca-1+). The percentages of LSK cells from RalA^Rosa26-Tg/+^ CML mice were higher than those from RalA^+/+^ mice *in vivo*. (G) BMCs were isolated from RalA^+/+^and RalA^Rosa26-Tg/+^ CML mice. A total of 1×10^5^ CML-like cells were plated on semisolid methylcellulose agar plates, and colonies were counted after 14 days. Scale bars, 100 µm. Colony formation results showed that RalA overexpression promoted the neoplastic capacity in CML.

In order to evaluate the *in vivo* role of RalA overexpression in CML, RalA^Rosa26-Tg/+^ and RalA^+/+^ BMCs were transfected using the BCR-ABL1-GFP retrovirus in line with the typical CML mouse model. BCR-ABL1-GFP-transfected RalA^Rosa26-Tg/+^ and RalA^+/+^ BMCs were injected into recipient mice under lethal irradiation (550 cGy, twice) (Figure 4A). RalA^Rosa26-Tg/+^ -BCR-ABL1 recipients died faster, and the median survival time (MST) was 11 days (n = 8) as compared to RalA^+/+^ recipients (MST, 16 days; n = 8; **P<0.01) (Figure 4B). RalA overexpression significantly promoted splenomegaly in CML mice in terms of volume (Figure 4C) and weight (Figure 4D). GFP+ (BCR-ABL-expressing leukemia cells) populations in BM increased significantly in the RalA^ Rosa26-Tg/+^ group (Figure 4E). Thus, the overexpression of RalA accelerates leukemogenesis *in vivo*.

### RalA overexpression accelerated CML development *in vivo*

To characterize the *in vivo* effect of RalA on regulating CML LSCs, we transduced BMCs in mice exposed to 5-FU, RalA^Rosa26-Tg/+^, RalA^+/+^, and the BCR-ABL1-GFP retrovirus. The abovementioned cells were then injected into recipient mice under lethal irradiation. Recipients of BMCs transformed with BCR-ABL from RalA^Rosa26-Tg/+^ donor mice (2.47%) showed significantly rapid CML development as compared to those receiving BMCs transformed using BCR-ABL from RalA^+/+^ donor mice (1.73%) (Figure 4F).

To determine the role of RalA in colony formation *in vivo*, RalA^Rosa26-Tg/+^ and RalA^+/+^ CML mouse BMCs were inoculated into semisolid agar plates, followed by colony counting after 14 days. Interestingly, following RalA overexpression, CML BMCs showed a greater capacity to form colonies than RalA^+/+^ CML BMCs (Figure 4G). The abovementioned findings indicate that RalA significantly promoted the development of BCR-ABL-mediated CML.

### RalA upregulation increased the self-renewal ability of LSCs *in vitro*

**Figure 5:**
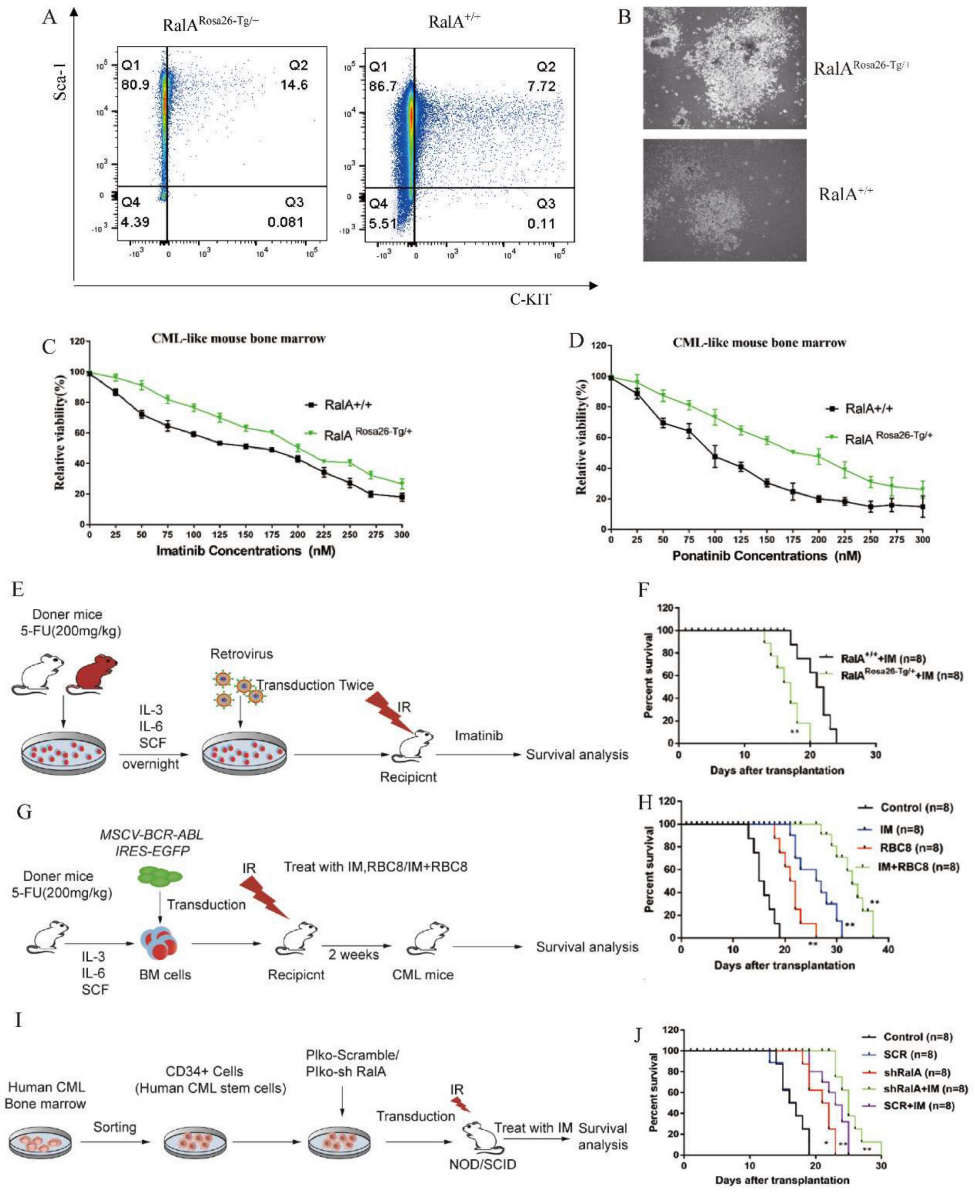
** RalA regulates CML development.** A. FACS analysis of LSK *in vitro*. BCR-ABL1-transformed CML-like cells from the BM of RalA^+/+^ and RalA^Rosa26-Tg/+^ mice were cultured under growth conditions of stem cells for 2 weeks after transduction. The cells were gated based on Lin- phenotype, and surface expression of Sca-1 and c-Kit (LSK) is shown. RalA increased the self-renewal ability of BCR-ABL-expressing LSK cells. (B) A total of 1×10^5^ RalA^+/+^and RalA^Rosa26-Tg/+^ BM cells were transduced with the BCR-ABL1 retrovirus and then plated on semisolid methylcellulose agar plates, and colonies were counted after 14 days. Scale bars, 100 µm. The results showed that RalA overexpression caused an increase in the colony-forming ability of BCR-ABL-expressing LSK cells. BM cells from RalA^+/+^ and RalA^Rosa26-Tg/+^ mice were transduced with the BCR-ABL1 retrovirus and then treated with different concentrations of imatinib (C) and ponatinib (D) (0-0.3 µM) for 48 h, followed by the determination of cell viability by MTT assay. RalA overexpression conferred resistance to imatinib and ponatinib *in vitro*. (E) Schematic of the BCR-ABL1-driven CML mouse model and imatinib treatment. (F) Kaplan-Meier survival curves for the recipients of BCR-ABL-transduced BM cells from the RalA ^Rosa26-Tg/+^ and RalA^+/+^ donor mice. All mice were treated with the same dose of imatinib. The median survival time (MST) was 16 days for the RalA^Rosa26-Tg/+^group (n = 8) and 22 days for the RalA^+/+^group (n = 8), **P<0.01. Mean ±SD values of three experiments and P-values are indicated. The results showed that RalA overexpression conferred resistance to imatinib *in vivo*. (G) Schematic of the BCR-ABL-driven CML mouse model and imatinib or RBC8 treatment. (H) Kaplan-Meier survival curves for the recipients of BCR-ABL-transduced BM cells from the RalA^+/+^ donor mice. All recipients of BCR-ABL-transduced BM cells from the RalA^+/+^ donor mice were treated with saline (black line), RBC8 (red line), imatinib (blue line), or RBC8+imatinib combination (green line). The median survival time (MST) was 15 days for the saline group (n = 8), 22 days for the RBC8 group (n = 8), 28 days for the imatinib group (n = 8), and 35 days for the RBC8+imatinib group (n = 8), **P<0.01. Mean ±SD values of three experiments and P-values are indicated. This result showed that targeting RalA by RBC8 exerted a strong antitumor effect *in vivo*. (I) Flowchart of evaluation of the *in vivo* effect of RalA knockdown on the lifetime of CML mice with shRalA. (J) Targeting RalA by shRNA exerted a strong antitumor effect on human CML CD34+ cell-driven mouse model. Survival of human CML CD34+ cell-driven NOD-SCID CML mice treated with scrRNA (n = 8), shRalA RNA (n = 8), imatinib+scrRNA (n = 8), and imatinib+shRalA RNA (n = 8) for 30 days, **P<0.01. Mean ±SD values of three experiments and P-values are indicated.

To clarify the role of RalA in the CML mouse model, we quantified the frequency of RalA^Rosa26-Tg/+^ and RalA^+/+^ CML LSCs *in vitro*. After transfection of BMCs using BCR-ABL1 with IL-3/IL-6/SCF, 14.6% of RalA^Rosa26-Tg/+^ CML-like cells retained the LSK phenotype. By contrast, the RalA^+/+^ CML-like cells with LSK phenotype accounted for only 7.72% (Figure 5A); this finding revealed that RalA overexpression in BMCs maintained an LSK cell phenotype.

Clone formation in cells is closely associated with their neoplastic ability. Therefore, the colony formation capacity was compared between RalA^Rosa26-Tg/+^ and RalA^+/+^ CML-like cells *in vitro* (Figure 5B). The colony size of RalA^Rosa26-Tg/+^ CML BMCs was larger than that of RalA^+/+^ CML BMCs; thus, demonstrating that RalA exerts a critical impact on the self-renewal ability of CML-initiating cells.

### FCM results suggested a remarkable increase in the number of myeloid (Gr-1^+^/Mac-1^+^) precursors in spleens and BM from RalA^Rosa26-Tg/+^ mice

We analyzed whether RalA overexpression decreased the production of mature myeloid and erythroid cells. Compared to mice in the RalA^+/+^ group, a significant number of mice in the RalA^Rosa26-Tg/+^ group showed expansion of myeloid leukemia cells (Gr-1+/Mac-1+) (Supplementary Figure S8). These findings suggested that RalA overexpression induces the expansion of mature myeloid cells and myeloid progenitors *in vivo*.

### RalA overexpression conferred imatinib resistance

Given that RalA overexpression shortens the lifespan of the CML mice model, we analyzed the role of RalA in regulating imatinib and ponatinib sensitivity in CML cells. As shown in Figure 5C, after imatinib treatment for 48 h, the IC_50_ values of RalA^Rosa26-Tg/+^ and RalA^+/+^ cells were 200 and 150 nmol/L, respectively. Furthermore, after ponatinib treatment for 48 h, the IC_50_ values of RalA^Rosa26-Tg/+^ and RalA^+/+^ cells were 175 and 110 nmol/L, respectively (Figure 5D). Taken together, these findings indicate that RalA overexpression led to a CML mouse model resistant to imatinib and ponatinib.

To assess whether RalA expression promotes imatinib resistance *in vivo*, BMCs from RalA^Rosa26-Tg/+^ and RalA^+/+^ mice were transduced to construct the mouse model of BCR-ABL-driven CML and then exposed to imatinib (100 mg/kg/day, gavage) (Figure 5E). Recipients of RalA-BCR-ABL died faster, and the MST was 16 days (n = 8) relative to the RalA^+/+^ group (MST, 22 days; n = 8; **P<0.01) (Figure 5F). Thus, RalA overexpression conferred *in vivo* and *in vitro* resistance to imatinib.

### RBC8 extended the survival of BCR-ABL1-driven CML mouse

To provide evidence supporting the *in vivo* pharmacological inhibitory effect of RalA on CML, a CML mouse model was adopted for testing the effects of exposure to the RalA inhibitor RBC8. A mouse model of human BCR-ABL1-driven CML was generated and then exposed to the RalA inhibitor RBC8 to evaluate its *in vivo* functions in CML. We randomized CML mice into 4 experimental groups and subjected them to a 14-day treatment period: normal saline solution, imatinib (100 mg/kg/day, gavage), RBC8 (50 mg/kg/day, gavage), and the corresponding combination treatment. Imatinib and RBC8 alone remarkably extended the survival of CML mice, and imatinib, when combined with RBC8, further prolonged the survival of CML mice (Figure 5G, H). Collectively, targeting RalA through RBC8 had a potent *in vivo* anticancer activity; furthermore, RalA could serve as a possible antileukemia target.

### Targeted inhibition of RalA suppressed xenograft tumor growth of human CML CD34+ cells in NOD/SCID mice

In order to further explore our hypothesis in primary progenitor cells, RalA expression was inhibited using shRalA or scramble RNA (SCR RNA) in primary CML CD34+ progenitor cells collected from eight independent CML cases. The cells were then engrafted into NOD/SCID mice for 30 days. The specific inhibition of RalA increased mouse survival, and imatinib treatment significantly prolonged the survival of CML mice under RalA knockdown condition (Figure 5I, J). Collectively, targeting RalA through shRNA potently suppressed the *in vivo* anticancer activity, while imatinib combined with RalA depletion treatment was effective on the CML CD34+ cell-driven mouse model.

### RalA interacted with RAC1 in cells and *in vitro*

**Figure 6:**
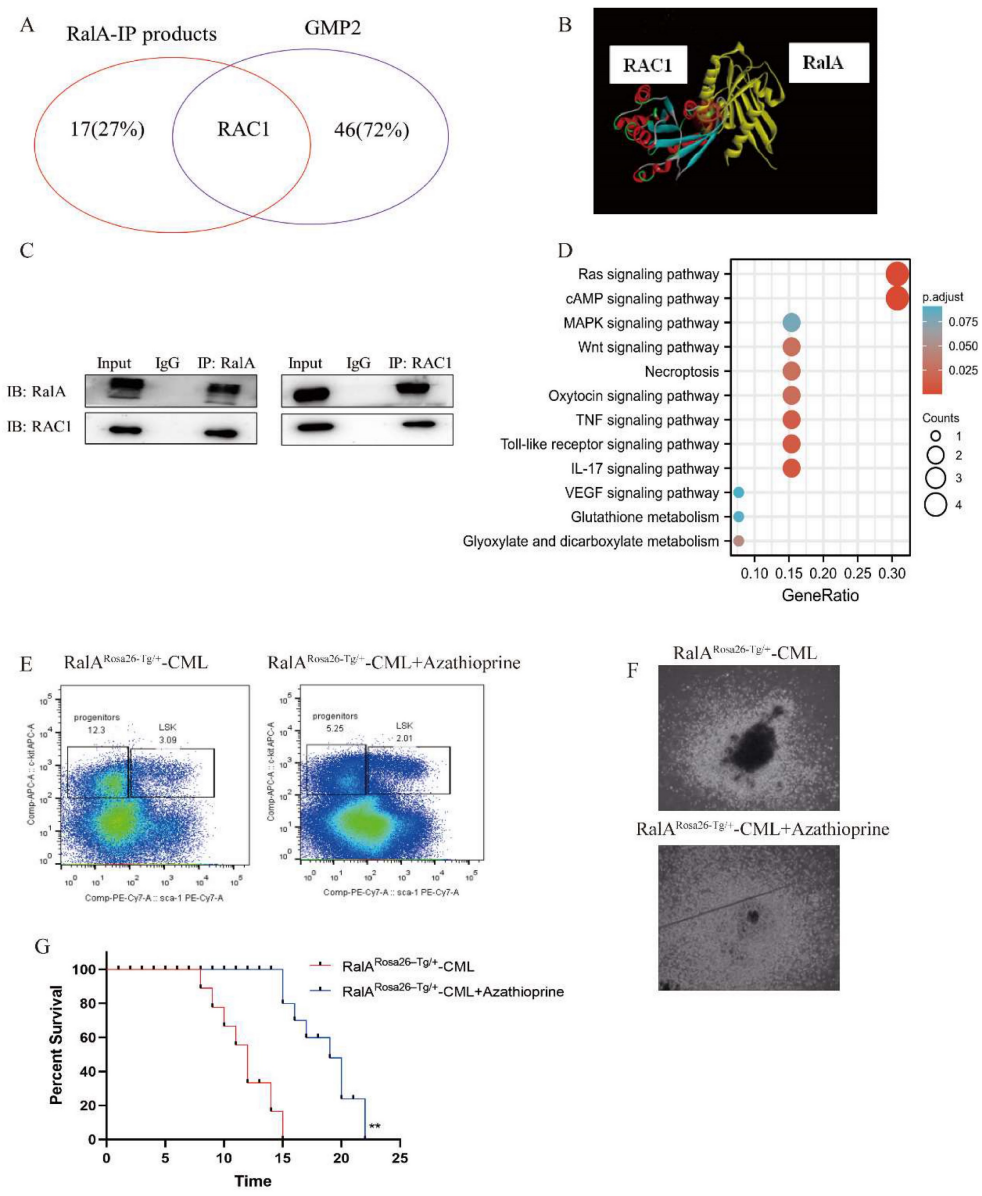
** RalA functions by binding to RAC1.** (A) 18 proteins were identified as the direct interacting proteins with RalA in K562 cells, while 47 genes were identified as GMP-specific genes in RalA^Rosa26-Tg/+^ mice; among these genes, only RAC1 was annotated as the gene associated with RalA. (B) Molecular docking of RalA (yellow)-RAC1(multicolour) determined using the ligand and receptor model in Discovery Studio 4.5. (C) Endogenous RalA and RAC1 interaction as determined by immunoprecipitation (IP) assays. K562 cells (1 × 10^7^ cells) were lysed with the transmembrane protein extraction reagent and antirabbit immunoglobulin G (IgG) or anti-RalA/RAC1 antibodies, followed by western blotting assay of RalA and RAC1. The results showed that RalA interacted with RAC1. (D) Signaling pathways determined using the integration of the signaling molecules from RalA IP products by KEGG analysis. RalA IP products are involved in the Ras-associated Wnt and MAPK signaling pathways. (E) RAC1 inhibition suppresses the self-renewal capacity of CML LSCs. RalA^+/+^ mice were transduced with the BCR-ABL retrovirus for 15 days, and the mice were divided into two groups (n = 8 biologically independent samples per group). One group received azathioprine, and the other group received normal saline and was used as the control group. After 14 days, the number of GFP+LSK cells in the BM of the mice was examined by FACS. (F) RAC1 inhibition drastically reduced the colony formation ability of the BCR-ABL-driven CML BMCs. RalA^+/+^ mice received BCR-ABL retroviral transduction and were divided into two groups (n = 6, biologically independent samples per group). One group received azathioprine, and the other group received normal saline and was used as the control group. The colonies were counted on day 14. Scale bars, 100 µm. (G) Azathioprine could prolong the survival rate in RalA overexpression CML mice. n = 8, **P<0.01. Mean ±SD values of three experiments and P-values are indicated.

To determine the RalA-related mechanism involved in CML, we performed immunoprecipitation (IP)-2D nano-HPLC-MS/MS assay using the CML-derived K562 cells. We identified several CML-associated proteins from the purified precipitates, which were later separated using RalA antibodies. Among these proteins, RAC1 attracted our attention as it is a member of the RAS family, and previous studies have revealed that it could bind to RalA. *RAC1* was also identified as a GMP-specific gene in the RalA overexpression group through single-cell RNA-Seq assay (Figure 6A); thus, RAC1 may share a close association with RalA. A molecular docking assay was performed using Discovery Studio. According to the “Receptor-Ligand” interaction result, RalA interacted with RAC1 (Figure 6B). This result was also confirmed in K562 cells by western blotting assay, in which RAC1 was immunoprecipitated by RalA, and conversely, RalA was immunoprecipitated by RAC1 in K562 cells (Figure 6C). These findings indicated that RalA might interact with RAC1 in CML cells.

### RalA participated in the Wnt and MAPK signaling pathways as determined by KEGG analysis

To determine the signaling pathways of RalA-related proteins, we investigated the signaling pathways based on the results of the anti-RalA immunoprecipitates obtained using KEGG integrated pathway analysis. PYCR2, GLUL, RAC1, MAP1A, RRM2, FAT1, PITPNB, SMCHD1, FOS, ADAR, ADRM1, ROCK2, RABGAP1L, TNFAIP3, RREB1, DAPK2, and PTPRC were present in the RalA immunoprecipitates. KEGG results revealed that RalA participates in the Ras, cAMP, MAPK, Necroptosis, Oxytocin TNF, Toll-like receptor, IL-17, VEGF, Glutathione metabolism, and Glyoxylate and dicarboxylate metabolism signaling pathways (Figure 6D).

### RAC1 inhibition suppressed the self-renewal capacity of CML LSCs and prolonged the survival rate in CML mice

To validate the pharmacological activity of RAC1 in suppressing CML *in vivo*, a CML mouse model of BM transduction/transplantation (BMT) was used. Next, 2.5 mg/kg azathioprine was administered to CML mice for a 14-day period, which markedly reduced the population of LSK cells (Lin-Sca-1^+^c-Kit^+^) from 3.09% to 2.01% (Figure 6E). Colony formation ability was then assessed in azathioprine-treated CML mice and controls, and the results showed that azathioprine dramatically reduced the colony formation ability of the BCR-ABL-driven CML BMCs (Figure 6F). We also found azathioprine could significantly prolong the survival rate in RalA overexpression mice, After azathioprine treatment the MST of RalA^Rosa26-Tg/+^ were 19 days (n = 8) relative to the control group (MST, 15 days; n = 8; **P<0.01) (Figure 6G). These results supported the role of RAC1 to be a functional target of RalA in CML cells.

## Discussion

The self-renewal capability of HSCs induces the generation of diverse blood lineages and allows the long-term generation of blood cells 24. However, it remains unclear how HSCs balance differentiation and self-renewal. More knowledge on the regulation of the abovementioned processes will help to formulate measures for improving HSC utilization in clinical settings and provide information for treating hematopoietic malignant diseases 25, 26. RalA is an important molecule in the RAS downstream pathway, and overexpression of oncogenic RAS in hematopoietic cells causes myeloproliferative disorder (MDS) 27. Nevertheless, the effects of RalA activation on the regulation of HSCs and leukemic cells are less well understood. By using a combination of single-cell technology and FCM, RalA was detected to be a vital factor that regulates the self-renewal of LSCs and HSCs.

With advances in single-cell technologies, the drawbacks related to hematopoietic cell populations defined by phenotypes have been overcome 28, 29. According to our findings, scRNA-seq revealed that RalA overexpression led to the expansion of HSC, MPP, and GMP cells; this finding is consistent with the results of surface marker-based immunophenotypes in RalA^ Rosa26-Tg/+^mice. The heterogeneity of the 2 HSC and 3 GMP populations was color-coded according to cell subclusters with a distinct molecular signature. We found that oxidative phosphorylation is involved in the RalA-induced expansion of HSCs. In previous studies, HSCs were found to mainly utilize glycolysis and derive energy through mitochondrial oxidative phosphorylation to meet the growing energy demand needed for differentiation 30. In GMP cells, *S100a8* and* S100a9* are the two important regulatory molecules, and previous studies have shown that inducing the expression of *S100a8-S100a9* causes a defect in p53-mediated erythroid differentiation 31. *S100a8-S100a9* has been detected to be a regulator of myeloid differentiation in leukemic cells, and it can be used to treat monocytic and myelomonocytic AMLs 32, 33. *CD52* is a unique gene in GMP2 and has critical effects on MDS 34. As shown in Figure 3F, HIF signaling was enriched in GMP2, and HIF is necessary to maintain the survival of CML CSCs. HIF-1α is an important pathway in LSCs, and the inhibition of the HIF-1α pathway can offer a treatment strategy to eliminate LSCs in CML 35. These findings reveal that RalA may promote GMPs toward leukemia. The single-cell analysis provides a further understanding of hematopoiesis and highlights the complexity of hematopoietic cell differentiation 36. Thus, we confirmed that RalA promotes the self-renewal of HSCs and GMPs.

The persistent activation of growth regulatory pathways through the Ras protein is the primary concern in CML, and CML CSCs can achieve this proliferation ability. Unfortunately, to date, Ras has not been identified as a druggable target; thus, more efforts have been focused on the Ras protein superfamily. RalA is one of the two proteins in the Ral protein family of the Ras branch of small GTPases 37. RalA activation has a critical effect on Ras-mediated human cell carcinogenesis 17, and this protein has been suggested to participate in the development of diverse cancers 38 such as melanoma 39, colorectal cancer 40, and lung cancer 41.

Our previous study showed that RalA acts as an important oncogene in CML 19. *RalA* also acts as a direct target gene for miR181a, a prognostic marker of CML 42. The RalA pathway is an important link during Ras-mediated carcinogenesis and transformation, and thus, RalA might also have a critical effect on CML oncogenesis. Nonetheless, the precise effect of RalA on CML pathogenesis and its effects on imatinib activity remain unknown. The current work analyzed the effects of RalA on CML development by using conditional RalA knockdown mice. RalA overexpression decreased the survival of BCR-ABL1-driven CML mice as compared to WT mice. In the present study, we used mice models overexpressing RalA to demonstrate that RalA had a critical effect on CML occurrence and LSC maintenance.

LSCs are an important cause of treatment failure in patients with CML. A previous study showed that RalA expression obviously elevated in CD34+ CML cells 43. Ral GTPases are responsible for Wnt signaling-mediated intestinal stem cell growth and renewal 44; this result revealed that RalA might have a close relationship with CML CSCs. RalA also promoted the self-renewal of LSCs.

RBC8, a RalA inhibitor 45, shows high selectivity for RalA and RalB GTPases, without inhibiting additional small GTPases including RhoA and Ras. To demonstrate the effect of RalA on CML, RBC8 was used to treat the CML mouse model. The results revealed that exposure to RBC8 effectively prolonged the survival of CML mice driven by BCR-ABL1; furthermore, the combination of RBC8 with imatinib markedly prolonged the survival of CML mice.

The activated RalA can bind to effector proteins for mediating related pathways 46, 47. Here, we observed that Rac1 interacted with RalA in CML through direct protein interaction, similar to that observed for insulin-dependent glucose absorption in muscle cells 48. Rac1 and Rac2 affect xenograft growth in stem cell niche, stem cell survival, and cell cycle and retention in the microenvironment of the normal hematopoietic process 49. Furthermore, as reported in a previous study, Rac1 activation promoted FGFR1-mediated leukemia occurrence in the stem cell leukemia syndrome, while Rac1 promoted HSPC expansion in initiating and maintaining leukemia in the mouse model 50. Rac1/2 activation can enhance FGFR1-triggered leukemia occurrence in stem cell leukemia/lymphoma syndrome 51. Our results are in accordance with the abovementioned findings. Furthermore, the inhibition of Rac1 by azathioprine effectively reduced the self-renewal ability of LSCs in the mouse model of BCR-ABL1-driven CML, and the colony formation ability was also inhibited by azathioprine.

## Conclusions

The findings of the current study suggested that RalA GTPase influences the expansion and self-renewal of normal and malignant HSPCs in a mouse model constructed with CRISPR/Cas9 gene editing technology. The high expression of RalA possibly contributes to the malignant transformation that facilitates the development of BCR-ABL1-mediated leukemic disease and imatinib resistance in mice. The potential molecular mechanism involves RalA, which is a therapeutic target for CML, and its interaction with RAC1. Collectively, these results help to understand the RalA-related mechanism in normal HSPCs and LSCs, and RalA could serve as a possible therapeutic target for eliminating CML LSCs.

## Supplementary Material

Supplementary figures.

## Abbreviations

HSPCs: hematopoietic stem and progenitor cells
CML: chronic myelogenous leukemia
LSCs: leukemic stem cells
HSC: hematopoietic stem cell
GEF: guanine nucleotide exchange factor
BM: bone marrow
GRB2: growth factor receptor-bound protein 2
scRNA-seq: single-cell RNA sequencing
sgRNAs: single-guide RNAs
i.p.: intraperitoneally
APC: allophycocyanin
IMDM: Iscove's modified Dulbecco's medium
IRES: internal ribosome entry site
MSCV: murine stem cell virus
SCF: stem cell factor
GFP: enhanced green fluorescent protein
WT: wild type
SDS: sodium dodecyl sulfate
5-FU: 5-fluorouracil
HRP: horseradish peroxidase
PBS: phosphate buffered saline
MTT: 3-(4,5-dimethylthiazol-2-yl)-2,5-diphenyltetrazolium bromide
MPP: multipotent progenitors
KEGG: Kyoto Encyclopedia of Genes and Genomes
LSK: (Lin-Sca1+c-kit+)
LT-HSC: long-term HSC
GMP: granulocyte-macrophage progenitors
UMAP: uniform manifold approximation and projection
MST: median survival time
IP: immunoprecipitation
BMT: BM transduction/transplantation
MDS: myeloproliferative disorder
ESCs: embryonic stem cells
PCR: polymerase chain reaction
