# Supplementary material for: Single-cell resolution reveals RalA GTPase expanding hematopoietic stem cells and facilitating of BCR-ABL1-driven leukemogenesis in a CRISPR/Cas9 gene editing mouse model
Source: Int J Biol Sci. 2023 Feb 13;19(4):1211–27. doi: 10.7150/ijbs.76993 (PMC10008703; doi:10.7150/ijbs.76993)
Supplement: Supplementary file 1 — Supplementary figures. [file ijbsv19p1211s1.pdf]

Supplementary information

A

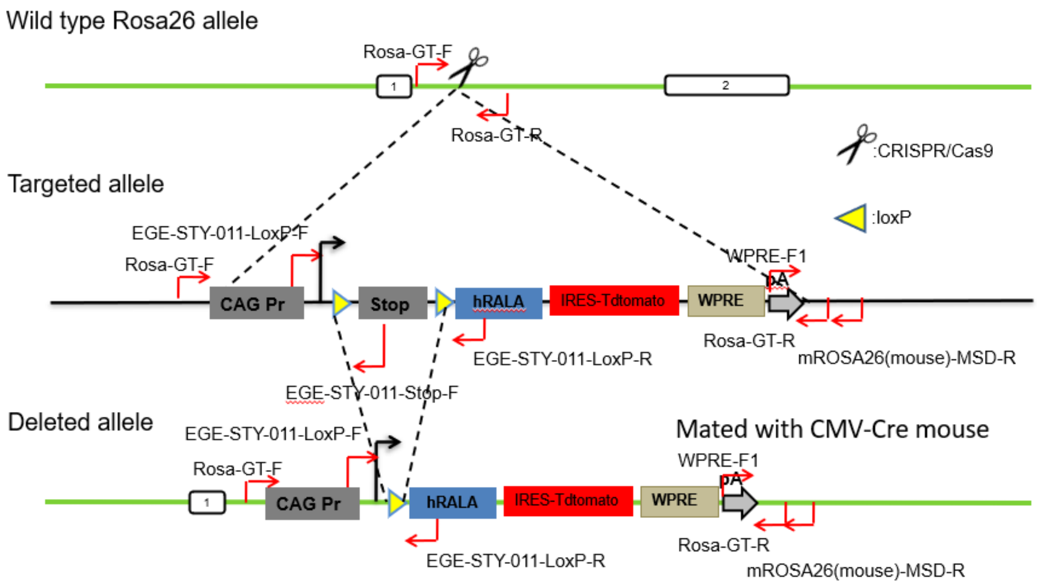

B

Primer	Sequence	Tm(℃)	Product size(bp)
Rosa-GT-F	AGTCGCTCTGAGTTGTTATCAG	56	WT:469<br>Mut:7265
Rosa-GT-R	TGAGCATGTCTTTAATCTACCTCGATG	58	
WPRE-F1	GCATCGATACCGTCGACCTC	58	Mut: 792
mROSA26(mouse)-MSD-R	TTTGATAAGGCTGCAGAAGGAGCGG	62	
EGE-STY-011-LoxP-F	CAACGTGCTGGTTATTGTGCTG	61	Mut: 212<br>WT: 1649
EGE-STY-011-LoxP-R	GCCCACCATGATGACTTTGTG	59	
EGE-STY-011-Loxp-F	CAACGTGCTGGTTATTGTGCTG	63	Mut: 304
EGE-STY-011-Stop-R	GCAACAACCGTCGTGGTGGT	64	
Cre-F4	GCGATCGCTGCCAGGATATACG	61	Mut:385
Cre-R4	CCAGAGTCATCCTTAGCGCCGT	62	

Supplementary Figure S1. Gene editing, identifying strategy and Primer information for genotyping

A. Gene editing, identifying strategy. B. Primer information for genotyping

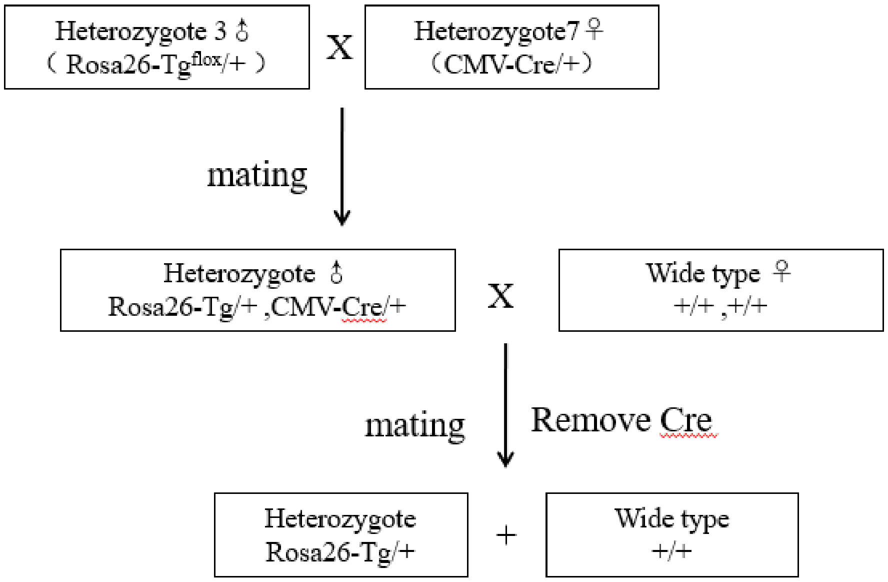

Supplementary Figure S2. Mating scheme to get the mice for CML mouse model

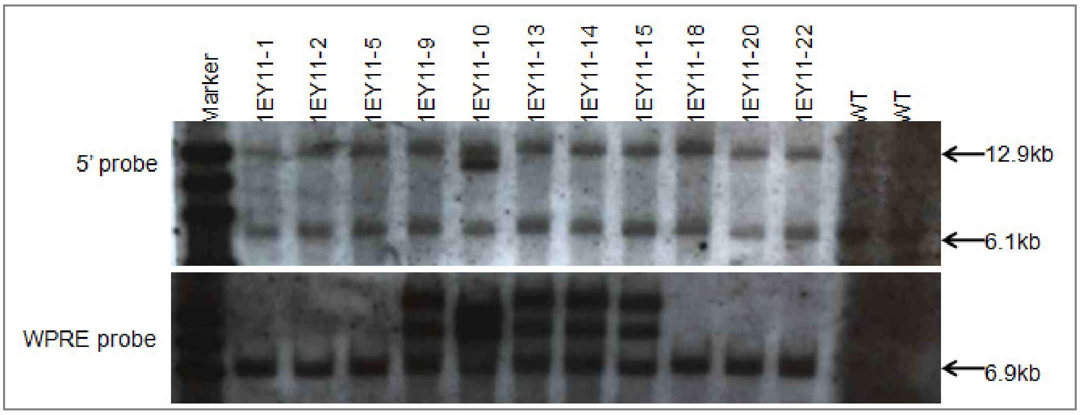

Supplementary Figure S3. Northern blotting results of F1 generation mice

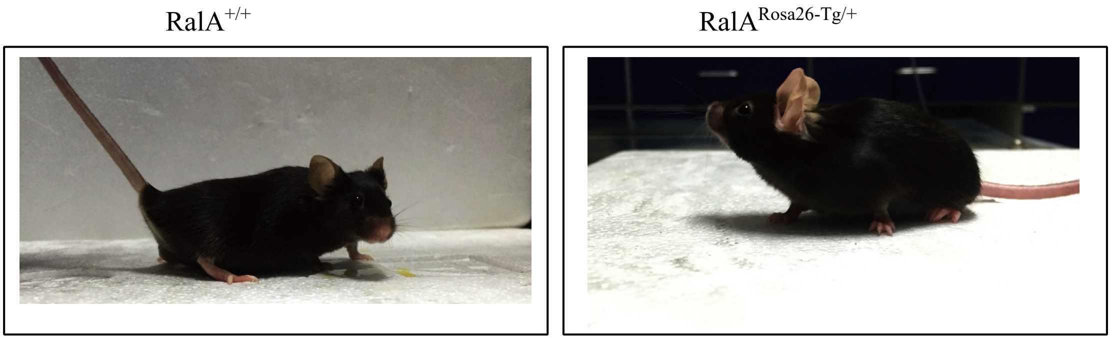

Supplementary Figure S4. The growth status of RalA$^{+/+}$ and RalA$^{Rosa26\text{-}Tg/+}$ mice

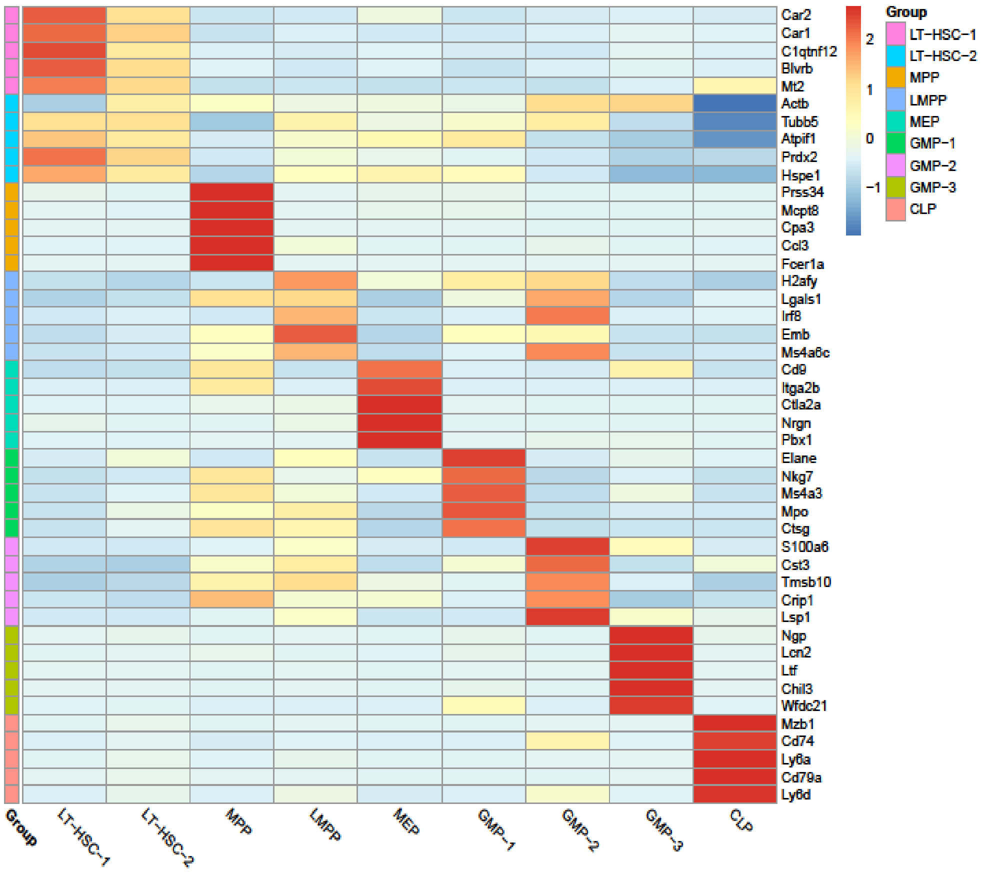

Supplementary Figure S5. Heatmap showing the expression of top 5 enriched genes in each cluster. Expression values are scaled (z-scored) for visualization

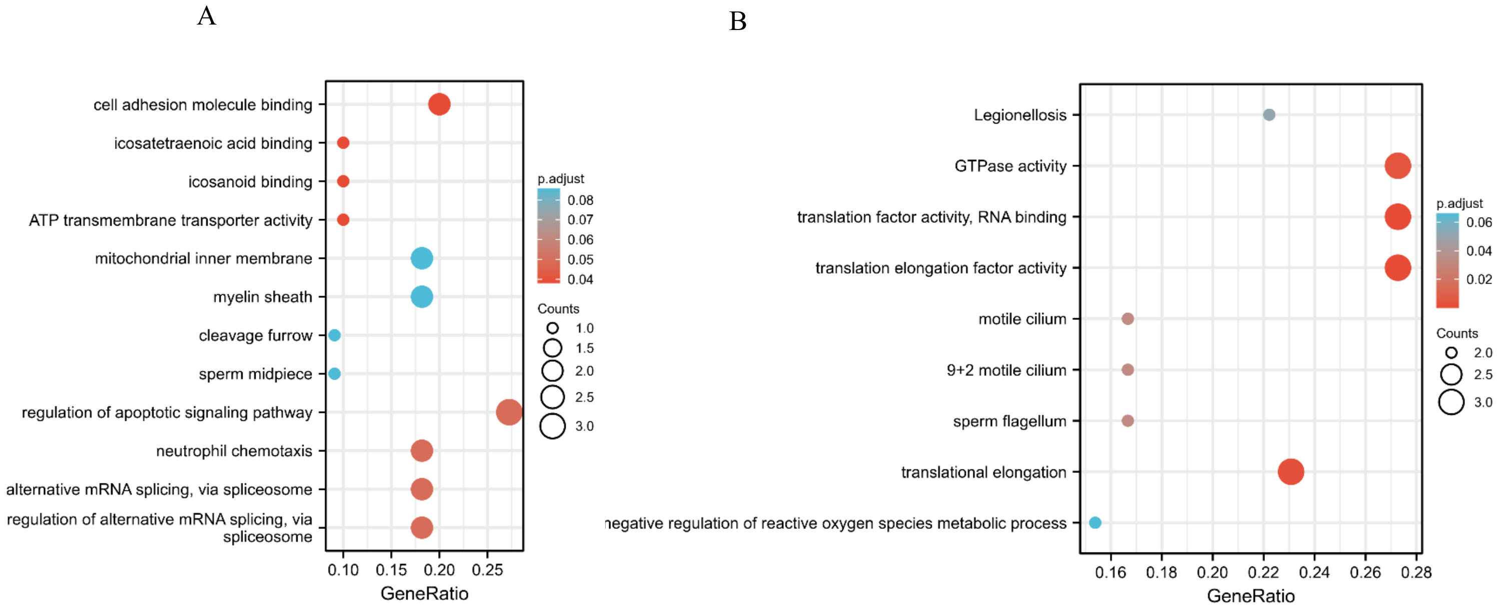

Supplementary Figure S6. Pathway enrichment analysis of the top 10 up-/down-regulated genes from GMP2 (A) and GMP3 (B), respectively.

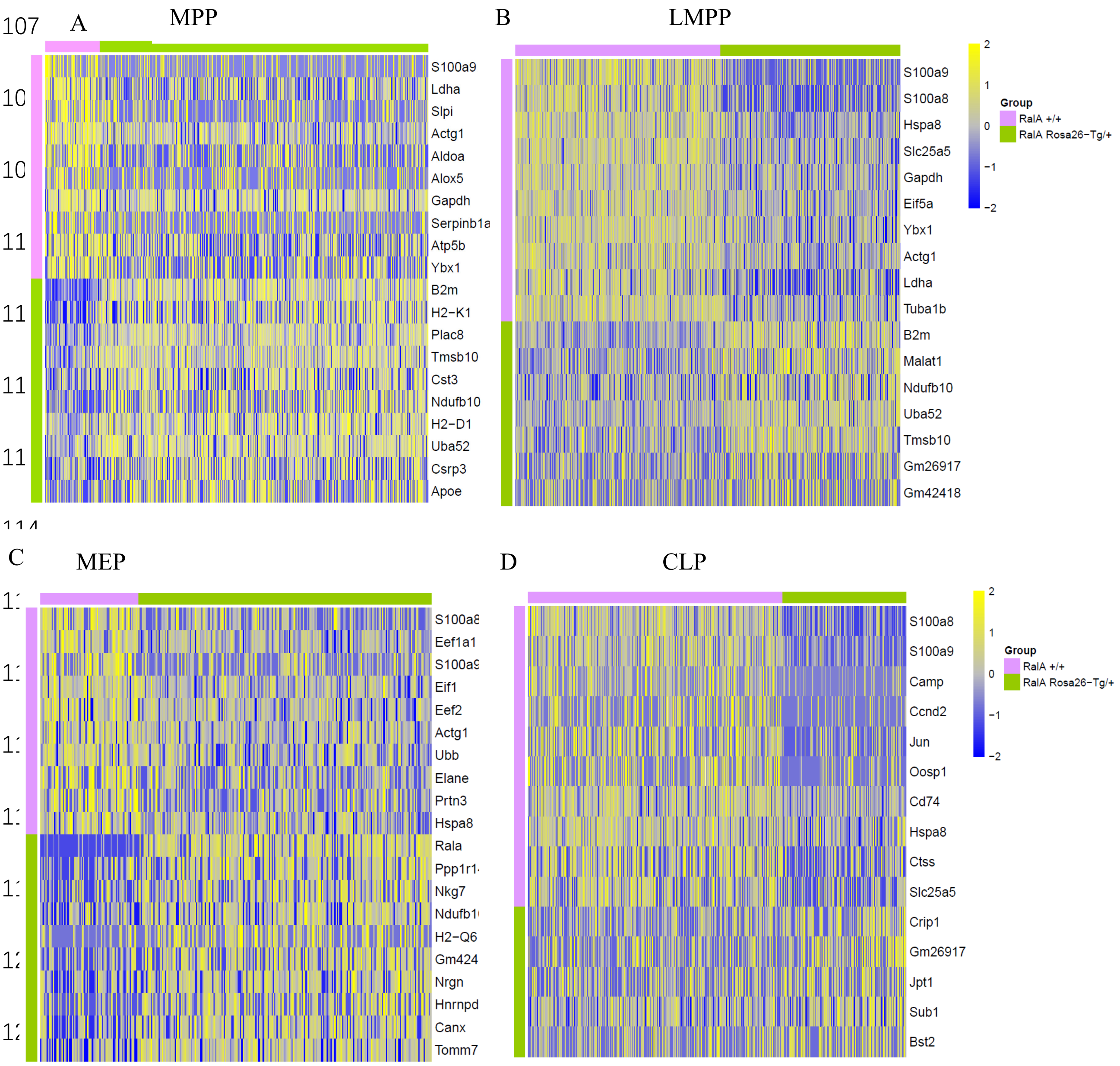

Supplementary Figure S7. The top 10 up or down-regulated genes enriched in MPP (A, left), LMPP (B, right), MEP (C, left) and CLP (D, right) clusters are displayed in the heat map.

A (Bone marrow)

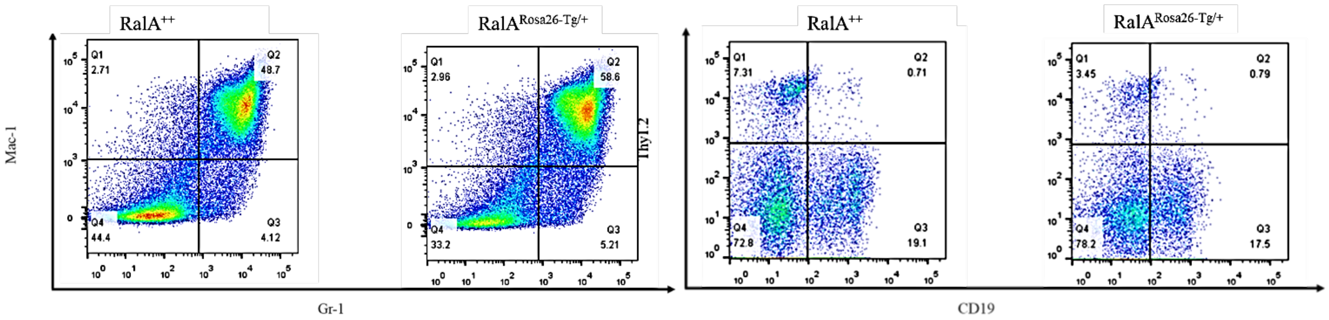

B (Peripheral Blood)

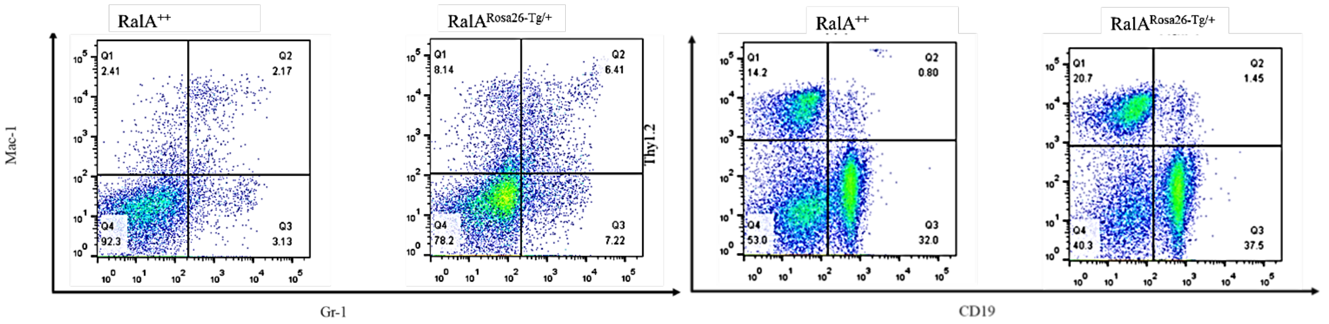

C (Spleen)

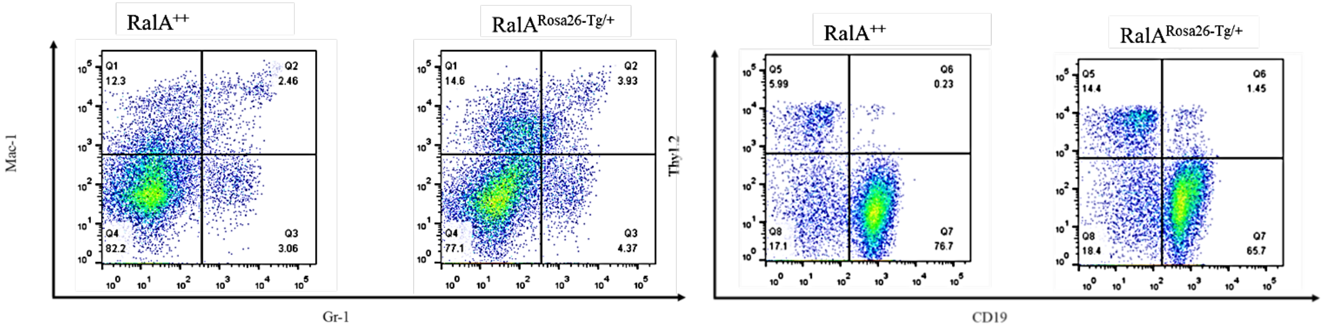

Supplementary Figure S8. RalA induces cell differentiation in some organs. BM (A), PB (B) and Spleen (C) cells were collected from RalA$^{+/+}$ and RalA$^{Rosa26\text{-}Tg/+}$ mice and stained with antibodies against Mac1, Gr1, CD19, or Thy1.2 analyzed by FACS. RalA overexpression promotes the percentage of myeloid cells in bone marrow, peripheral blood, and spleen in RalA$^{Rosa26\text{-}Tg/+}$ mice.
